# A Case of Poisoning with Oil of Tansy

**Published:** 1852-11

**Authors:** John C. Dalton


*A Case of Poisoning with Oil of Tansy—Death at the end of three hours and a half—Quantity of the Drug taken about* ℥j *and* ʒiii. By John C. Dalton, Jr., M. D. (Read to the Boston Society for Medical Observation, June 2, 1851.)—E. S., a fine, healthy-looking girl, about twenty-one years of age, died at the house of Mr. A., in Boston, on Wednesday, the 7th of May, 1851. She had been employed in Mr. A.'s family as a seamstress since the previous winter, living in the house during the week, but going away on Saturdays to a cousin's in Pleasant street, and returning to Mr. A·' son Monday morning. She had been, for some months, receiving the attentions of a young man who was reputed to be engaged to her. None of her friends, however, suspected anything to be wrong with her until Monday, May 5, when her cousin, with whom she had been spending Sunday, as usual, perceived the odor of tansy in the room which she had occupied; whereupon it occurred to her that the girl might have become pregnant, and used the drug for the purpose of producing abortion.

On Tuesday, she was engaged in her ordinary employment, and dined heartily a little after five o'clock in the afternoon. She went up stairs to her room about half-past nine o'clock. The cook, who occupied a room above, went up with her and stopped in her room, conversing for some fifteen minutes. The girl's manner was perfectly natural and cheerful, as it had been throughout the day. About a quarter before ten o'clock the cook left her preparing for bed, and went up to her own room.

Nothing more was heard from her till about eleven, when Mr. and Mrs. A., who were sitting in the basement-room, heard a scream, which they supposed to come from one of the children. Mrs. A. went immediately up stairs, and on entering Miss S.'s room, found her on the floor, by the side of the bed, insensible and in violent convulsions. She had evidently fallen out of bed, as she was undressed, and the bedclothes were disturbed, and had been partially dragged on the floor with her. Dr. Morrill was immediately sent for, and arrived in about ten minutes. He sent also for me, and I arrived at the house at half-past eleven o'clock.

The girl was then lying on her back by the side of her bed, and presented the following appearances: Total unconsciousness; cheeks flushed; of a bright, red color; eyes open and very brilliant; pupils of equal size; widely dilated and immovable; sclerotics injected; skin warm, not remarkable as to moisture; respiration hurried, labored, stertorous, and obstructed by an abundance of frothy mucus, which filled the air-passages, and was blown from between the lips in expiration; the breath had a strong odor of tansy, as had been already observed by Dr. Morrill; pulse quite full, forcible, 128; at intervals of five to ten minutes the body was convulsed by strong spasms, in which the head was thrown back, the respiration suspended, the arms raised and kept rigidly extended, and the fingers contracted. After this state of rigidity had continued for about half a minute, it was usually succeeded by a tremulous motion, often sufficient to shake the room, together with very faint and imperfect attempts at inspiration. The whole interval from the commencement of the convulsion to the first full inspiration, varied from a minute to a minute and a-half. Occasionally, the tongue was wounded by the teeth, and the saliva slightly tinged with blood. Immediately after a convulsion the countenance was very pallid and livid, from the suspension of respiration, and the pulse exceedingly reduced in strength and frequency. The pulse and color then gradually returned until the occurrence of the next spasm. It was very common, a few seconds after the termination of a convulsion, for the head to be drawn slowly backward, and the eyelids, at the same time, stretched wide open. In the intervals of the convulsions the limbs were mostly relaxed, but the jaws remained clenched.

A vein was immediately opened in the right arm, and about Oij of blood taken away. After this, the pulse became much softer, and the face lost its bright color. There was, however, no change in the condition of the pupils, nor return of consciousness, nor other improvement in the appearance of the patient. It being impossible to get anything down the throat, two injections of an ounce of wine of antimony, with about ʒss of powdered ipecac., were thrown up the rectum at intervals of about half an hour, but produced no apparent effect.

On searching the room, a ℥ij phial was found in the pocket of the girl's dress, wrapped in a piece of paper, labelled "Oil of Tansy," and marked with the name and address of an apothecary in Pleasant street. The phial contained ʒv of the oil of tansy of the ordinary purity.—A mug was also found from which she had apparently drunk the oil, mixed with water, as it smelt very strongly of the drug, and still had a drop or two of it at the bottom.

The condition of the patient continued much the same for about an hour. The convulsions, however, gradually became less protracted, and the failure of the pulse after each attack, more complete, at the same time that it recovered strength less perfectly in the intervals. The countenance also became somewhat sunken and the temperature of the skin reduced. About 1 o'clock, six leeches were applied to the forehead and temples, and sinapisms put on the calves of the legs. The leech-bites bled freely.

Toward two o'clock the alteration for the worse became quite rapid. Pulse 124 and feeble; respiration 36, and attended with less muscular effort than at first; the left cornea was glazed, but the right continued brilliant; a little inward strabismus of the right eye, and the mouth and nose drawn a little to the right side. Occasionally a slow, lateral, rolling motion of the eyeballs. At five minutes past two she had the last convulsion, which was much less violent than the earlier ones, and lasted only half a minute. There was no recovery of the pulse after this attack, and she died at a quarter past 2 o'clock, A. M.

The present case is another instance of the extreme violence to which the system may be subjected even in the early months of pregnancy, without inducing abortion. Though all the muscles, both of the body and limbs, were for three hours and a quarter subjected to a succession of the most violent contractions, there was no sign of abortion, and after death the ovum was found in the uterus entirely undisturbed. In Dr. Hildreth's case, also, pregnancy existed but a few weeks advanced, and the drug was undoubtedly taken for the purpose of producing abortion, but nothing of the kind took place. The general symptoms in that case were similar to those described in the foregoing, the most remarkable difference being the more gradual loss of consciousness, and the more rapid death after a much smaller dose.—*American Jour. Med. Sciences.*

